# A Service Evaluation of Eating Disorders Training for Psychiatry Trainees in Wales

**DOI:** 10.1192/bjo.2025.10489

**Published:** 2025-06-20

**Authors:** Umer Jalal, Nermeen Ahmed, Isabella Jurewicz, Hafeesa Sameem, Manjula Simiyon

**Affiliations:** 1 Swansea Bay University Health Board, Swansea, United Kingdom; 2 Cardiff and Vale University Health Board, Cardiff, United Kingdom; 3 Betsi Cadwaladr University Health Board, Wrexham, United Kingdom

## Abstract

**Aims:** Concerns about the inadequacies in eating disorders (EDs) training are widely acknowledged. Through this comprehensive project, we aim to delve deeper in identifying knowledge and training gaps regarding EDs in Wales. The specific aims are: to ascertain gaps in understanding of EDs amongst psychiatry trainees in Wales, and to evaluate the current teaching and training offered to them.

**Methods:** Between 7 and 14 October 2024, two cross-sectional, Microsoft web surveys were distributed among core trainees’ year 3 (CT3s) and higher trainees (HTs) in psychiatry across Wales. Participation was voluntary and anonymised. Surveys included seven Likert-scale questions and one free-text question. Postgraduate teaching and training on EDs between August 2023 and August 2024 by Health Education and Improvement Wales (HEIW) and the six university health boards (UHBs) offering psychiatry training were also surveyed.

**Results:** A total of 28 HTs and 13 CT3s completed the surveys. Over 60% of trainees reported low confidence (rated 5 and below) in describing various EDs, their prevalence, and risk profiles. Only 50% of HTs felt confident (rated 6–10) diagnosing EDs compared with 69% of CT3s. Additionally, only 57% of HTs felt confident in communicating with people with EDs and assessing their needs, compared with 77% of CT3s. Furthermore, 75% of HTs felt unsure about the stages and types of EDs management compared with 54% of core trainees. On the other hand, 85% of CT3s and 68% of HTs felt confident in describing medical emergencies in EDs.

Above 80% of trainees expressed dissatisfaction with education and training provided. No ED-related postgraduate teachings or specific placements were offered across most UHBs during the review year. Exposure to ED patients was primarily through Child and Adolescent Mental Health Services placements, with limited opportunities based on trainees’ interest. HEIW offered one teaching session on EDs to each CT1 and CT2/3 cohorts; however, the CT1 session was cancelled. Since 2023, HEIW has been funding the Royal College of Psychiatrists’ ED credential for interested HTs in Wales.

**Conclusion:** The noticeable gaps in trainees’ understanding and training in EDs highlight the urgent need for improved educational and training programs. To effectively address these gaps, gaining insight into trainees’ perspectives and working collaboratively with trainers can lead to the development of more effective training strategies.

